# The diffuse-type tenosynovial giant cell tumor (dt-TGCT) patient journey: a prospective multicenter study

**DOI:** 10.1186/s13023-021-01820-6

**Published:** 2021-04-29

**Authors:** Nicholas M. Bernthal, Geert Spierenburg, John H. Healey, Emanuela Palmerini, Sebastian Bauer, TOPP Study Group, Bart Schreuder, Andreas Leithner, Javier Martin-Broto, Francois Gouin, Thomas Cosker, Hans Gelderblom, Eric L. Staals, Julio Lopez-Bastida, Eva-Maria Fronk, Xin Ye, Petra Laeis, Michiel A. J. van de Sande

**Affiliations:** 1 Division of Musculoskeletal Oncology, David Geffen School of Medicine at UCLA, Santa Monica, CA USA; 2 Department of Orthopaedic Surgery, Leiden University Medical Center, Leiden, The Netherlands; 3 Department of Surgery, Orthopaedic Surgery Service, Memorial Sloan Kettering Cancer Center, New York, NY USA; 4 Medical Oncology, Musculoskeletal Oncology Department, IRCCS Istituto Ortopedico Rizzoli, Bologna, Italy; 5 Department of Medical Oncology, Sarcoma Center, West German Cancer Center, University of Duisburg-Essen, Essen, Germany; 6 Leiden University Medical Center, Leiden, The Netherlands; 7 Department of Medical Oncology, Leiden University Medical Center, Leiden, The Netherlands; 8 Department of Orthopaedic Surgery, Musculoskeletal Oncology Department, IRCCS Istituto Ortopedico Rizzoli, Bologna, Italy; 9 Faculty of Health Sciences, University of Castilla-La Mancha, Talavera de la Reina, Toledo Spain; 10 Daiichi Sankyo Europe GmbH, Munich, Germany; 11 Daiichi Sankyo, Inc., Basking Ridge, NJ USA

**Keywords:** Arthroscopy, Diagnosis, Patient journey, Surgery, Synovectomy, Systemic therapies, Diffuse TGCT, TOPP registry

## Abstract

**Background:**

Tenosynovial giant cell tumor (TGCT) is a rare, locally aggressive neoplasm arising from the synovium of joints, bursae, and tendon sheaths affecting small and large joints. It represents a wide spectrum ranging from minimally symptomatic to massively debilitating. Most findings to date are mainly from small, retrospective case series, and thus the morbidity and actual impact of this rare disease remain to be elucidated. This study prospectively explores the management of TGCT in tertiary sarcoma centers.

**Methods:**

The TGCT Observational Platform Project registry was a multinational, multicenter, prospective observational study involving 12 tertiary sarcoma centers in 7 European countries, and 2 US sites. This study enrolled for 2 years all consecutive ≥ 18 years old patients, with histologically diagnosed primary or recurrent cases of diffuse-type TGCT. Patient demographic and clinical characteristics were collected at baseline and every 6 months for 24 months. Quality of life questionnaires (PROMIS-PF and EQ-5D) were also administered at the same time-points. Here we report baseline patient characteristics.

**Results:**

166 patients were enrolled between November 2016 and March 2019. Baseline characteristics were: mean age 44 years (mean age at disease onset: 39 years), 139/166 (83.7%) had prior treatment, 71/166 patients (42.8%) had ≥ 1 recurrence after treatment of their primary tumor, 76/136 (55.9%) visited a medical specialist ≥ 5 times, 66/116 (56.9%) missed work in the 24 months prior to baseline, and 17/166 (11.6%) changed employment status or retired prematurely due to disease burden. Prior treatment consisted of surgery (i.e., arthroscopic, open synovectomy) (128/166; 77.1%) and systemic treatments (52/166; 31.3%) with imatinib (19/52; 36.5%) or pexidartinib (27/52; 51.9%). Treatment strategies at baseline visits consisted mainly of watchful waiting (81/166; 48.8%), surgery (41/166; 24.7%), or targeted systemic therapy (37/166; 22.3%). Patients indicated for treatment reported more impairment compared to patients indicated for watchful waiting: worst stiffness NRS 5.16/3.44, worst pain NRS 6.13/5.03, PROMIS-PF 39.48/43.85, and EQ-5D VAS 66.54/71.85.

**Conclusion:**

This study confirms that diffuse-type TGCT can highly impact quality of life. A prospective observational registry in rare disease is feasible and can be a tool to collect curated-population reflective data in orphan diseases.

*Name of registry*: Tenosynovial Giant Cell Tumors (TGCT) Observational Platform Project (TOPP).

*Trial registration number*: NCT02948088.

*Date of registration*: 10 October 2016.

*URL of Trial registry record*: https://clinicaltrials.gov/ct2/show/NCT02948088?term=NCT02948088&draw=2.

**Supplementary Information:**

The online version contains supplementary material available at 10.1186/s13023-021-01820-6.

## Introduction

Tenosynovial giant cell tumor (TGCT) is a rare, locally aggressive mesenchymal neoplasm arising from the synovium of joints, bursae, and tendon sheaths and affects both small and large joints [1]. Two main subtypes of TGCT are defined based on clinical and radiological characteristics: localized- and diffuse-type TGCT (L-TGCT and dt-TGCT). The malignant version of TGCT is extremely rare [2]. From the molecular point of view, both subtypes usually share the presence of a fusion involving the colony stimulating factor (CSF) gene, which drives tumor growth [3, 4]. Although both subtypes share a common pathophysiology, they represent a wide spectrum of clinical entities, making TGCT behavior complex and hard to predict [5]. Clinical disease spectrum ranges from mildly symptomatic to extremely debilitating, where patients present with symptoms like pain, stiffness, swelling, and limitation in range of motion [6]. Further characterization of disease severity has been made to identify cases as mild localized, severe localized, moderate diffuse, or severe diffuse [7]. Uniform magnetic resonance (MR) descriptions are of utmost significance for clinical and research purposes. The classification of clear MR criteria is challenging, due to the rarity of the tumor and small number of heterogeneous cases, variety of joints involved, different disease severity as well as several treatment modalities. To date, MR imaging (MRI) has shown to be the best distinguishing method for evaluation of TGCT. The proposed TGCT severity classification informs physicians and patients on disease extent and risk for recurrence after surgical treatment. Definition of the most severe subgroup attributes to a universal identification of eligible patients for systemic therapy or trials for novel agents [7]**.** Also, ultrasound has been used effectively in the evaluation of soft-tissue masses, particularly involving the knee joint. Soft-tissue sarcomas appear as a complex mass with increased vascularity [8]. Diffusion-weighted echo-planar MRI is potentially helpful in differentiating malignant soft tissue tumors from benign masses as well as in grading malignancy [9]. Muscle sarcomas are indicated to present a broad range of apparent diffusion coefficient (ADC), dependent on diffusion-weighted imaging (DWI) which is crucial regarding tumor composition, and distinguishing between malignant and benign lesions [10]**.**

Although less prevalent, dt-TGCT is an aggressive multi-lobulated lesion located intra- and/or extra-articular, affecting various joints in the body (mainly the knee) and having a detrimental effect on quality of life (QoL) [11–14]. Incidence rate of dt-TGCT is estimated at 5 per million person-years [11]. Due to non-specific symptoms and the rarity of this disease, a proper diagnosis can sometimes take many years, which in turn may severely delay optimal treatment and care for these patients, resulting in them facing a higher risk of excessive, inadequate, or under treatment [11, 15]. Once diagnosed, treatment options include mostly surgical intervention. However, recently tyrosine kinase inhibitors (TKIs) that target the CSF1 receptor (CSF1R) have been used for treatment in cases where surgery is not an option [3, 16–21]

As the predominant epidemiologic understanding of dt-TGCT to date comes mainly from small, retrospective studies that traditionally focused on oncological outcomes, questions to elaborate the true morbidity and actual impact on QoL, both the disease and its various treatment options remains to be elucidated [1, 22]. Given this context, there is a need for a better understanding of the natural history of this tumor to understand the burden of dt-TGCT from a patient perspective and of the treatment landscape beyond a single institution. Additionally, there is the need to explore the current management of TGCT, particularly of the diffuse type (including functional details measured pre- and post-treatment) to describe the spectrum of indications, challenges, and the actual impact on patient QoL and ability to work.

To this end, the first multinational, multicenter, prospective, non-interventional, observational disease registry study, named TGCT Observational Platform Project (TOPP), was launched in November 2016, involving hospitals and tertiary sarcoma centers from Europe (EU) and the United States (US). All patients included in the study were to be followed up with for a minimum of 2 years. Herein, we report on patient demographics and clinical characteristics at the time when patients were entered into the registry (baseline). This includes main disease characteristics, treatment patterns, and outcomes of the dt-TGCT patient population from varying geographical regions to better understand the breadth of the patient journey. In addition, we aimed at identifying and describing factors influencing treatment decision making, in the absence of consensus treatment guidelines.

## Methods

### Study design and participants

This global multicenter, prospective sponsored study included all consecutive patients from 12 tertiary sarcoma centers in 7 EU countries from 2016 to 2018. Two sites in the US enrolled patients from 2017 to 2019. Patients were enrolled during a 2-year period with prospective follow-up over 24 months. Participating sites were selected based of their expertise in treatment of TGCT.

Eligible patients were 18 years or older, with a primary or recurrent dt-TGCT. TGCT had to be histologically confirmed and assessed as diffuse-type based on MRI or clinical presentation if this was missing. Dt-TGCT is often characterized by a multi-nodular tumor on MRI. The institutional review board or ethics board provided approval in each center, and written informed consent was obtained from each patient who participated in this study.

Primary diagnosis was defined as patients who were awaiting treatment or were treated and showed no evidence of local progression at baseline. Recurrent disease was defined as tumor recurrence after complete resection or progression of residual tumor. Therapy-naïve patients received no therapy prior to baseline and were consequently admitted as primary diagnosed patients. Disease severity was in line with the TGCT severity classification by Mastboom et al., with severe dt-TGCT classified as intra- and extra-articular involvement with involvement of one or more ligaments or muscular/tendinous tissue observed on MRI [7].

Patient demographics, complete TGCT-related history, and current status, including radiologic assessments and health resources used in the past 24 months, were collected at baseline. Baseline visits occurred at the outpatient clinic of either the department of orthopedic surgery or the oncology department. Baseline data on TGCT-related patient-reported outcomes (PRO) for pain, stiffness, swelling, and limitations in range of motion were collected and followed every year thereafter through electronic data capture. The patient-reported outcome measurements (PROMs) were administered at baseline consisting of the mean brief pain inventory (BPI), mean worst pain and stiffness numerical rating scale (NRS), the Patient-Reported Outcome Measurement Information System Physical Functioning® (PROMIS-PF), and the EuroQoL 5D (EQ-5D) (Additonal file 1). Admission status at baseline was categorized into patients with a primary diagnosis or recurrent disease.

### Statistical analysis

Continuous data were described using either means and standard deviations (SD) or medians and interquartile ranges (IQR). Categorical variables were summarized as number of observations and percentages (%) of the observations in each category. Percentages do not include the missing category and are calculated over the number of subjects with available (non-missing) data. The whole analysis was descriptive only. Statistical analysis was performed using the Statistical Analysis System (SAS©) Version 9.4 under Microsoft Windows Operating System. Because dt-TGCT is an orphan disease, no formal sample size consideration has been performed, as recruitment of patients within the scheduled 2-year period was expected to be difficult.

## Results

Between November 2016 and March 2019, 166 patients from the EU and US were enrolled in the TOPP registry. Description of baseline patient demographics and clinical characteristics are provided in Table 1. The mean age at diagnosis was 39.0 years (range, 14.4–75.6; SD ± 14.42) and median time from diagnosis until TOPP entry point was 29.7 months (IQR, 9.5–80.0). TGCT had a female predilection (n = 102; 61.4%), and the knee joint was predominantly affected (n = 112; 68.5%). Other involved locations were the ankle (n = 19; 11.4%), the hip (n = 12; 7.2%), the shoulder (n = 8; 4.8%), the foot (n = 5, 3.0%), the elbow (n = 3, 1.8%), the hand (n = 3, 1.8%), and the temporomandibular joint (n = 1; 0.6%). Ninety-five patients (57.2%) were primary diagnosed cases, and 71 patients (42.8%) had at least one recurrence prior to baseline, occurring after any treatment of their primary tumor.

**Table 1:** Demographic and clinical characteristics of patients included in the TOPP study at baseline

Features	n = 166 (%)
Mean age [years] at diagnosis ± SD	39.0 ± 14.42
Mean age [years] at baseline ± SD	44.0 ± 14.12
Female, n (%)	102 (61.4)
Level of education (n = 143)	
University (bachelor or higher)	63 (44.1)
Time [months] since diagnosis, median (Q1, Q3)	29.7 (9.5–80.0)
Localization, n (%)	
Knee	112 (68.5)
Ankle	19 (11.4)
Hip	12 (7.2)
Shoulder	8 (4.8)
Foot	5 (3.0)
Elbow	3 (1.8)
Wrist	3 (1.8)
Hand	3 (1.8)
Temporomandibular	1 (0.6)
Therapy prior to baseline, n (%)	139 (83.7)
Recurrent disease, n (%)	71 (42.8)
1 recurrence	37 (52.9)
2 recurrence	15 (21.4)
3 recurrence	18 (25.7)

*Q1* quarter 1, *Q3* quarter 3, *SD* standard deviation, *TGCT* tenosynovial giant cell tumor, *TOPP* TGCT Observation Platform Project

### Diagnostic pathway

A median of 16.9 months (IQR, 4.0–44.0) elapsed from onset of symptoms until diagnosis of TGCT (Table 2). Most commonly, MRIs requested closest to baseline of TOPP were for postoperative follow-up (n = 56; 40.0%). Of all MRIs, dt-TGCT was generally located both intra- and extra-articular (n = 90/147; 61.2%) with involvement of ligaments (n = 88/134; 65.7%), and tendons and muscles (n = 99/141; 70.2%), classifying half of the patients (n = 83) with severe dt-TGCT at baseline (Table 2). If assessable, severe dt-TGCT was observed in the knee, ankle, hip, and other locations in 51.5% (n = 51/99), 55.6% (n = 5/9), 58.8% (n = 10/17) and 77.2% (n = 17/22) of the cases, respectively.

**Table 2:** Diagnostic pathway (%)

Time [months] from onset symptoms until diagnosis, median (Q1, Q3)	16.9 (4.0–44.0)
*Information on MRI*	
Any closest^a^ to BL MRI, n (%)	157 (94.6)
Indication of MRI closest to BL, n (%)	
Primary diagnosis	36 (25.7)
Pre-surgery	16 (11.4)
Regular postoperative follow-up	56 (40.0)
Follow-up due to complaints	32 (22.9)
Missing	17
Characteristics of MRI, n (%)	
Both intra- and extra-articular (n = 147)	90 (61.2)
Extra-articular tendon/muscle involvement (n = 141)	99 (70.2)
Ligament involvement (n = 134)	88 (65.7)
TGCT severity, n (%)	
Moderate diffuse	64 (38.6)
Severe diffuse	83 (50.0)
Not assessable	19 (11.4)
*Information on biopsy*	
Any biopsy prior BL^b^ (restricted to the 95 patients with primary diagnosis), n (%)	86 (90.5)
Excisional biopsy	32 (41.6)
Core needle biopsy	14 (18.2)
Arthroscopic biopsy	11 (14.3)
Surgery for suspected cancer diagnosis	10 (13.0)
Fine needle aspiration biopsy	6 (7.8)
Other	9 (11.7)
Missing	9

*BL* baseline, *MRI* magnetic resonance imaging, *Q1* quarter 1, *Q3* quarter 3, *TGCT* tenosynovial giant cell tumor

^a^Defined as MRI with nearest date to Baseline visit date, with the date of MRI either before or equal to the Baseline visit date or—if no treatment yet performed—at the latest 30 days after the Baseline visit date

^b^Percentage calculation can sum to > 100% because patients can fall in more than one category

Sixty-nine patients (41.6%) were classified severe dt-TGCT even after treatment, exemplifying the continued severity of the disease. Histological confirmation was primarily obtained after excisional biopsy (n = 32; 41.6%), however several non-excisional biopsy techniques were also performed in other patients (e.g., core needle biopsy, arthroscopic biopsy, or fine needle aspiration). In 13%, TGCT diagnosis was based on surgical histology from samples obtained during procedure undertaken for suspicion of a malignancy (Table 2).

### Treatments received prior to baseline of TOPP

Of 166 patients who entered the TOPP study, 139 (83.7%) had already been exposed to a TGCT-related treatment, whereas only 27/166 patients (16.3%) were treatment-naïve (Table 1). Ninety-five patients (57.2%) were primary diagnosed cases, and 71 patients (42.8%) had at least one recurrence prior to baseline, occurring after any treatment of their primary tumor (Table 3).

**Table 3:** TGCT-related therapies prior to baseline, N (%)

	Tumor status		Total (n = 166)
	Primary diagnosis (n = 95)	Recurrent diseases (n = 71)	
Any surgery prior to baseline	57 (60.0)	71 (100)	128 (77.1)
Type of surgery prior to BL (if any)^a^			
Arthroscopic synovectomy	30 (31.6)	33 (46.5)	63 (49.2)
One-stage synovectomy	22 (23.2)	42 (59.2)	64 (50.0)
Two-stage synovectomy	6 (6.3)	7 (9.6)	13 (10.2)
(Tumor) prosthesis	1 (1.1)	4 (5.6)	5 (3.9)
Any systemic treatment prior BL	24 (25.3)	28 (39.4)	52 (31.3)
Type of last systemic treatment prior BL (if any)			
Tyrosine kinase inhibitors	22 (91.7)	25 (89.3)	47 (90.4)
Monoclonal antibodies	1 (4.2)	3 (10.7)	4 (7.7)
Other	1 (4.2)	–	1 (1.9)
Duration [days] until BL, Median (Q1, Q3)	307.00 (120.00–421.00)	186.00 (88.00–345.00)	236.00 (118.00–366.00)
Ongoing	11 (45.8)	7 (25.0)	18 (34.6)
Possible side effects	11 (45.8)	19 (67.8)	30 (58.8)
Any radiation therapy	5 (5.3)	10 (14.1)	15 (9.0)
Type of radiation therapy prior to BL (if any)			
Radiotherapy	2 (40.0)	4 (40.0)	6 (40.0)
^ 90^Yttrium	3 (60.0)	6 (60.0)	9 (60.0)
No prior therapy	27 (28.4)	–	27 (16.3)
Prior and concomitant therapies for TGCT-related symptoms	50 (52.6)	38 (53.5)	88 (53.0)

*BL* baseline, *Q1* quarter 1, *Q3* quarter 3, *TGCT* tenosynovial giant cell tumor

^a^Sum of all therapies can be more than total because a patient could have received ≥ 1 therapies

Of 57 patients treated with surgery at the time of initial diagnosis, 30 (31.6%) had been treated arthroscopically. At the time of relapse, 71 (100%) patients had a re-operation, and in this case the surgical approach was open synovectomy in 49 (69.0%) and arthroscopic in 33 (46.5%). Five patients (3.9%) had received a (tumor) prosthesis secondary to TGCT in four cases due to a recurrent tumor. Fifty-two patients (31.3%) received systemic treatment; in 39.4% (28/71) this was indicated in recurrent cases and was still ongoing in 34.6% (18/52) at baseline. Thirty-two of 52 cases (62.7%) were indicated for systemic therapies because of locally advanced TGCT, 9.8% (5/52) as neo-adjuvant, 7.8% (4/52) for maintenance, and 7.8% (4/52) for palliative therapy. Eleven patients (21.2%) received systemic therapies as first treatment for TGCT. TKIs imatinib (off label) or pexidartinib (in research setting) were most frequently administered as latest treatment prior to baseline (46/47; 97.9%) (Table 3). Radiation therapy, comprising external beam radiotherapy and radiosynoviorthesis with ^90^Yttrium, was administered in 15/166 (9%) and mostly performed as adjuvant therapy after surgery in refractory cases (10/15; 66.7%) (Table 3). Eighty-eight (53%) of all cases had received prior and concomitant therapies for TGCT-related symptoms.

### Treatment strategies at time of TOPP study entry

Treatment strategies at baseline visits of TOPP consisted of watchful waiting (n = 81/166; 48.8%), surgery only (n = 41/166; 24.7%), or targeted systemic therapy only (n = 37/166; 22.3%). A multimodality approach was administered in 7/166 (4.2%) of cases, comprising different therapy combinations (e.g., surgery, targeted systemic therapies, and/or radiation therapy) (Additonal file 1).

A conservative monitoring approach at baseline was primarily decided on for patients who received only surgery before baseline (n = 47/81; 58.0%) (Table 4). Most MRIs were conducted as regular postoperative follow-up (n = 43/75; 57.3%), and this group comprised the lowest percentage of severe cases (n = 38/81; 46.9%). Non-invasive interventions were common in this group; 26.2% of the patients received rehabilitation (n = 17), and patients in need of physical therapy (n = 23, 28.4%) had a median of 18 (range, 4.0–200.0) sessions.

**Table 4:** Patients’ presentation and reported outcomes at baseline by treatment strategy, N (%)

	Wait and See (n = 81)	Surgery only (n = 41)	Systemic only (n = 37)
Mean age [years] ± SD	44.3 ± 15.17	41.8 ± 14.94	47.7 ± 10.44
Time since diagnosis primary tumor [months] median (Q1, Q3)	34.3 (13.8–77.9)	6.7 (1.2–59.8)	32.1 (18.2–89.6)
Treatment before baseline			
Therapy-naïve	11 (13.6)	16 (39.0)	–
Surgery only	47 (58.0)	20 (48.8)	7 (18.9)
Systemic only	2 (2.5)	–	9 (24.3)
Multimodal treatment	21 (25.9)	5 (12.2)	21 (56.8)
Admission status			
Primary diagnosis	47 (58.0)	27 (65.9)	19 (51.4)
Recurrent diseases	34 (42.0)	14 (34.1)	18 (48.6)
Indication MRI closest to baseline			
Primary diagnosis	7 (9.3)	23 (57.5)	4 (11.4)
Pre-surgery	5 (6.7)	8 (20.0)	2 (5.7)
Regular postoperative follow-up	43 (57.3)	6 (15.0)	7 (20.0)
Follow-up due to complaints	15 (20.0)	2 (5.0)	13 (37.1)
Severity			
Moderate	34 (42.0)	15 (36.6)	12 (32.4)
Severe	38 (46.9)	20 (48.8)	21 (56.8)
Not assessable	9 (11.1)	6 (14.6)	4 (10.8)
In last 24 months prior to baseline			
Any rehabilitation	17 (26.2)	5 (13.2)	4 (12.5)
Specialist visits^a^, Median (range)	5.0 (1.0–70.0)	3.0 (10–27.0)	12 (2.0–65.0)
Physical therapy sessions^a^, Median (range)	18.0 (4.0–200.0)	11.0 (1.0–100.0)	11.5 (3.0–90.0)
Symptoms			
Pain	56 (69.1)	37 (90.2)	32 (86.5)
Stiffness	36 (44.4)	27 (65.9)	23 (62.2)
Swelling	44 (54.3)	34 (82.9)	19 (51.4)
Limited range of motion	39 (48.1)	31 (75.6)	30 (81.1)
≥ 3 symptoms	31 (38.3)	28 (68.3)	22 (59.5)
Analgesics use	8 (9.9)	5 (12.2)	9 (24.3)
Worst stiffness NRS Mean ± SD (n = 144)	3.4 ± 2.57	5.2 ± 3.14	5.3 ± 2.55
Worst pain NRS Mean ± SD (n = 81)	5.0 ± 2.41	6.5 ± 2.27	5.8 ± 1.97
Pain severity score Median (Q1, Q3) (n = 147)	2.25 (0.75–4.00)	4.25 (1.50–6.25)	4.25 (1.50–5.50)
Pain interference score Median (Q1, Q3) (n = 146)	1.57 (0.14–4.00)	3.00 (1.14–5.57)	3.00 (0.57–5.57)
PROMIS-PF Median (Q1, Q3) (n = 142)	44.43 (37.30–49.29)	39.54 (34.95–44.42)	39.98 (34.79–43.69)
EQ-5D Index score Median (Q1, Q3) (n = 153)	0.84 (0.67–0.89)	0.80 (0.53–0.84)	0.74 (0.48–0.84)
EQ-5D VAS Median (Q1, Q3) (n = 154)	79.0 (60.0–85.0)	69.0 (60.0–80.0)	70.0 (50.0–75.0)

*EQ-5D* EuroQol 5D, *MRI* magnetic resonance imaging, *NRS* numeric rating scale, *PROMIS* Patient-Reported Outcomes Measurement Information System; *PROMIS-PF* Patient-Reported Outcome Measurement Information System Physical Functioning®, *Q1* quarter 1, *Q3* quarter 3, *SD* standard deviation, *VAS* visual analog scale

^a^Based on patients that had any

Patients indicated for surgery in this population were most recently diagnosed with TGCT. A median of 6.7 (IQR, 1.2–59.8) months elapsed from TGCT diagnosis until baseline, and 65.9% (n = 27) had a primary diagnosis, of which 16/41 (39.0%) were therapy-naïve at baseline. Furthermore, MRIs closest to baseline were primarily indicated to diagnose TGCT (n = 23; 57.5%) (Table 4).

Twenty-one (56.8%) of the patients indicated for targeted systemic therapies at TOPP baseline already had received multimodality treatment before baseline. None of these patients were therapy-naïve at baseline, and just 7 (18.9%) patients had only surgery before. MRIs were predominantly obtained due to progressive complaints (n = 13; 37.1%), and in this patient group the highest percentage of recurrent (n = 18; 48.6%) and severe dt-TGCT (n = 21; 56.8%) was observed. These patients visited medical specialists at a median of 12 times (range, 2.0–65.0) in the 24 months prior to baseline. Patients indicated for systemic therapies had a median age of 48.0 years (range 20.0–73.0). In addition, analgesics were most used by these patients (n = 9; 23.3%) and mean worst stiffness and pain NRS scores of 5.3 (SD ± 2.55) and 5.8 (SD ± 1.97), respectively, were reported. Physical functioning was limited with a median PROMIS-PF score of 39.98, and the lowest QoL scores were reported with an EQ-5D index score of 0.74 and visual analog scale (VAS) score of 70.0. At baseline, 33 patients (89.2%) had a current systemic therapy, of which 18 (54.5%) were started before. All current systemic therapies consisted of TKIs imatinib (n = 14; 42.4%) and pexidartinib (n = 19; 57.6%).

Only 11 patients did not report complaints due to TGCT at baseline, resulting in 93.4% of patients with at least one complaint. Patients indicated for treatment reported TGCT-related symptoms (e.g., pain, stiffness, swelling, and limited range of motion) more frequently compared to those with a wait-and-see policy (Table 4), except for swelling, which was least experienced by patients treated with systemic therapies (51.4%), and 68.3% indicated for surgery at baseline suffered from 3 or more TGCT-related symptoms. Both patient groups indicated for surgery and systemic therapies reported higher pain severity (4.25) and interference scores (3.00) compared to patients indicated for watchful waiting (2.25; 1.57). In addition, both treatment groups reported lower PROMIS-PF scores (39.54 and 39.98, respectively), EQ-5D index scores (0.80 and 0.74, respectively) and EQ-5D VAS scores (69.0 and 70.0, respectively).

### Health economics related to the TOPP cohort

Thirty-three patients (23.9%) required at least 5 visits from disease onset, before reaching a diagnosis of TGCT. In addition, 76 patients (55.9%) consulted a medical specialist 5 times or more in the 24 months prior to baseline. Thirty-six patients (25.5%) had more than 10 physical therapy sessions in the 24 months prior to baseline. Hospitalization and rehabilitation were required in 91.0% (151/166) and 18.6% (26/140), respectively, with a median of 3.0 (range, 1.0–184.0) and 15.0 (range, 1.0–120.0) days, respectively. Fifteen (9.9%) patients were hospitalized 5 or more times. Sixty-six patients (56.9%) missed work due to their TGCT in the 2 years prior to baseline, with a median of 25.0 days (range, 1.0–75.0). More importantly, of 146 patients who were employed, 17 (11.6%) were forced to change their employment status or even retire prematurely due to disease burden. Domestic help was necessary in 26 cases (16.0%).

## Discussion

TOPP represents the largest prospective, international, multicenter disease registry for dt-TGCT, being able to include 166 patients in slightly more than 2 years and shows that conducting collaborative observational studies for a rare tumor is feasible. Current literature is largely focused on the oncological outcomes of this often-chronic disease [16, 18, 20, 23–27]. Baseline data derived from this registry help to describe a preliminary understanding of the dt-TGCT patient journey and treatment decisions around disease onset and diagnosis of dt-TGCT patients. We believe that such study design can guide collection of high-quality data for other orphan diseases.

**Fig. 1:**
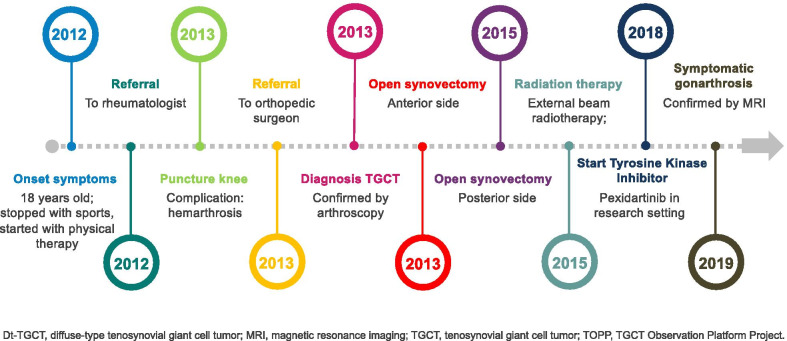
A typical timeline of dt-TGCT in a single TOPP patient. The disease had its onset in an 18-year-old patient who was forced to stop exercising and in need of physical therapy due to dt-TGCT-related complaints. Several recurrences occurred despite multimodality treatment, leading to secondary gonarthrosis at the age of 25

**Fig. 2:**
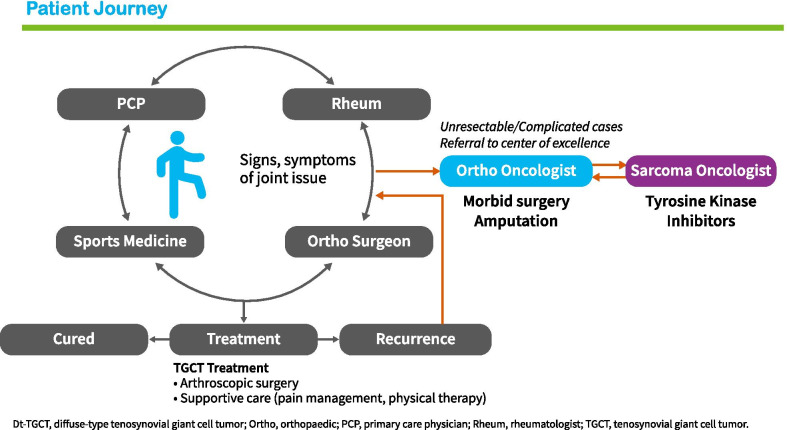
This figure represents the general patient journey of patients with dt-TGCT. Non-specific symptoms and disease unawareness results in several visits to different healthcare practitioners and unnecessary or excessive treatment in first and second line before referral to an orthopedic or sarcoma oncologist

The present study confirmed that TGCT has its onset in a relatively young, educated, and working patient population with a female predilection [11, 12]. Time between onset of symptoms until diagnosis averaged more than a year, and in this time interval several medical specialists were frequently visited. An under- or overestimation could be introduced due to a recall bias. Nonspecific clinical signs and symptoms in TGCT patients often mimicked other mono-articular pathologies, resulting in frequent consultation of various healthcare professionals (e.g.*,* physical therapists, rheumatologists, and sports doctors) and lag time in diagnosis (Figs. 1, 2) [28]. MRI was the non-invasive gold standard to diagnose TGCT type and distinguish between the localized and diffuse subtypes [29, 30]. In addition, this modality was frequently utilized for postoperative surveillance for recurrence, evaluation of worsening complaints (e.g., distinguishing degenerative arthritic symptoms or internal derangement of the joint), or pre-surgical planning (Table 2). Definitive diagnosis was predominantly obtained by histological confirmation through different forms of biopsies [31, 32]. In 10 cases, TGCT was coincidentally diagnosed after surgery for an initial suspicion of cancer. Disease mimicking and unfamiliarity could possibly introduce such misdiagnoses, with potential major consequences for a patient.

The primary form of care for TGCT is complete surgical removal of abnormal tissue, performed arthroscopically, open or combined, often requiring multiple incisions to access the disease thoroughly. However, there is a high risk for recurrence, especially in dt-TGCT, due to invasive growth both in and outside the joint [15, 19, 24, 33]. Synovectomies are generally relatively invasive, with a high recurrence rate and repetitive surgery causing significant impairment [24]. Multimodality treatments (e.g.*,* external beam radiotherapy and radiosynoviorthesis) have been performed in an attempt to reduce the recurrence rate in dt-TGCT, leading to varied reported outcomes [25–27]. In addition to surgery, several CSF1R inhibitors including TKIs showed promising results in tumor volume decrease and reduction of debilitating symptoms [16, 18, 20, 21, 34]. Of the TKIs, pexidartinib is an FDA-approved systemic therapy, recently added as a category 1 recommendation for the treatment of adult patients with symptomatic TGCT/pigmented villonodular synovitis (PVNS) associated with severe morbidity or functional limitations that is not amenable to improvement with surgery.

**Fig. 3:**
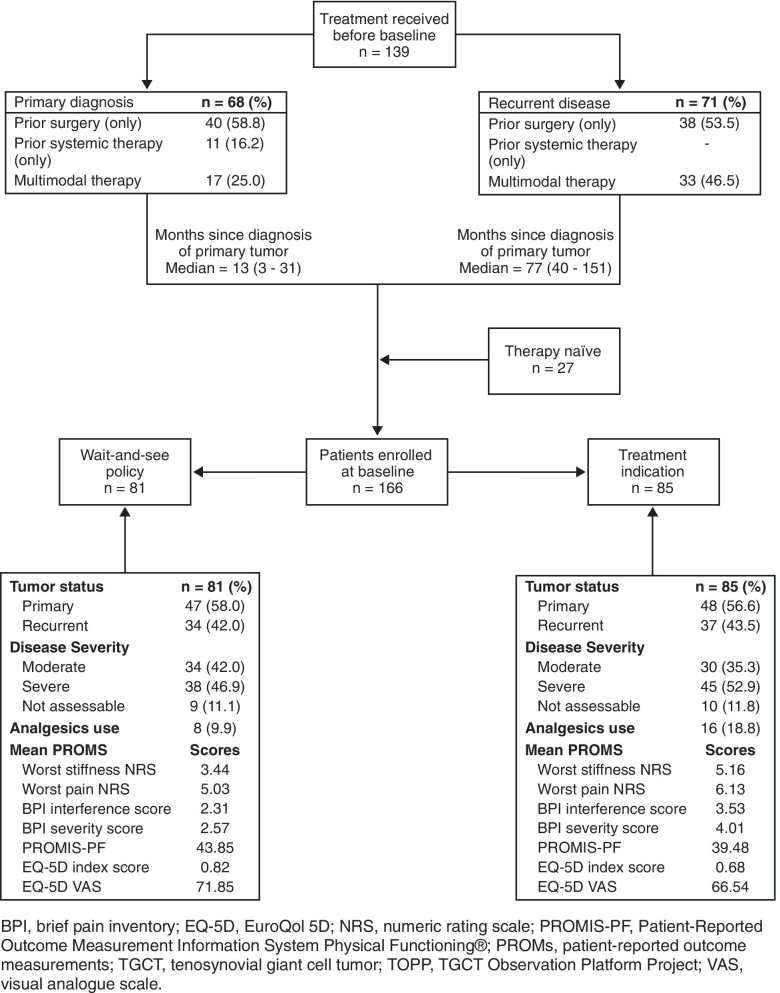
This flowchart gives a schematic overview of the treatment types patients received prior to TOPP, according to tumor status: primary diagnosis or recurrent disease. In addition, the cohort is stratified into 2 patient groups according to treatment plan at baseline: watchful waiting and indicated treatment at baseline. Possibly important factors in treatment decision making are shown per subgroup

Our results confirm that surgery was the mainstay of treatment (75%), which is consistent with other studies [15, 24, 33]. Furthermore, all patients with recurrent dt-TGCT disease had surgery, often combined with other treatment modalities (Fig. 3). Synovectomies were mostly performed open. To date, literature reported conflicting results regarding different surgical techniques, not favoring one over another [23, 35, 36]. However, we hypothesize that open surgery may allow for better overview of tumor, located intra- and extra-articular, with extension to surrounding tissues, possibly resulting in more complete removal of disease burden. Almost a third of the patients received systemic therapies, mainly TKIs such as pexidartinib (in research setting) and imatinib (off label)—a relatively high percentage, possibly due to a selection bias since sarcoma centers participating in TOPP were also involved in clinical studies on TGCT. Use of TKIs was mostly found indicated in locally advanced refractory cases, illustrating this modality being considered a last resort for patients who are not amenable for surgery (Fig. 2). An individual well-thought-out treatment decision made by a multidisciplinary team of medical specialists is therefore needed regarding both surgical and systemic treatment options with such rates of response, local recurrence, complications, and side effects.

Given the lack of understanding of this disease, the incidence of TGCT may be underestimated as disease awareness increases and diagnostic tools improve [13]. Diagnostic delay results in multiple visits to different health care practitioners (e.g.*,* general practitioner, physiotherapist, sports medicine doctor, rheumatologist) and unnecessary or excessive treatments (e.g.*,* use of painkillers or diagnostic arthroscopies) in the first and second line before referral to an orthopedic or sarcoma oncologist [15, 37]. If treated inadequately, aggressive dt-TGCT can become a chronic illness affecting an otherwise young, healthy patient population, leading to a significantly decreased QoL and concurrent high social costs (e.g.*,* sick leave, medical costs) (Fig. 1) [13, 38].

Current literature lacks treatment guidelines and does not present relevant clinical findings that support clinical decision making. Creating insight on such important factors can be of great value in optimizing treatment strategies. Different treatment strategies were selected at baseline of TOPP, predominantly watchful waiting, surgery, or systemic therapies. The number and type of follow-up visits were not controlled, as they were influenced by patient and physician concerns. Systemic therapies were predominantly indicated for older patients with recurrent and severe dt-TGCT despite their having received multimodality treatment before. This patient group reported the highest decrease in QoL and experienced a major limitation in physical functioning. The use of systemic therapies in the setting of relapsed dt-TGCT might be justified in an attempt to avoid chronic disability [16, 18, 20]. Local experience and availability of TKIs during TOPP possibly influenced the choice for treatment in the tertiary reference centers, with a preference for surgery followed by TKI. Primary or refractory cases are predominantly treated at doctors’ preference. Improved disease-specific patient education, multidisciplinary discussion, and shared decision making would enable better treatment selection for each patient.

At baseline of TOPP, patients with a wait-and-see policy reported fewer TGCT-related symptoms, less frequent use of painkillers, and higher QoL, advocating that the lack of symptoms may be the driving force for choosing a more conservative approach. We therefore considered PRO to be important influencers in shared treatment decision making, which is consistent with the increasing role of patient-based care in chronic diseases, especially in a benign disease such as TGCT [39].

The aim of TOPP is to provide insight on disease burden including healthcare utilization, treatment landscape, and current management of TGCT in the tertiary sarcoma center setting. In 2 years prior to baseline, medical professionals were often consulted, a fourth of the patients needed multiple physical therapy sessions, and medical specialists were frequently visited by more than half of the patients (Table 5). Hospitalization and, to a lesser degree, rehabilitation were common with varying duration. Like the study by Burton et al., this suggests that TGCT causes a high health economic burden. In a like manner, this suggests that dt-TGCT increases social costs [38]. In the population studied, illness often caused work absence, intermittently more than 5 weeks of work in total in 2 years’ time (Table 5). Additionally, several patients were forced to change their employment status from full-time to part-time, some had to become unemployed, while some even had to enter early retirement, all due to dt-TGCT. The demand for domestic help illustrates the impairment in activities of daily living. Worsening of dt-TGCT over time will potentially increase the interference of the disease with work and the healthcare utilization. However, since this study only reports data captured at baseline, we are not able to analyze such a change over a period of time.

**Table 5:** Health economics prior to baseline, N (%)

Any referral/specialists visits prior to diagnosis (n = 138)	135 (97.8)
≥ 5	33 (23.9)
24 months prior to baseline	
≥ 5 GP visits (n = 132)	21 (15.9)
≥ 5 specialists visits (n = 136)	76 (55.9)
≥ 10 PT sessions (n = 141)	36 (25.5)
Rehabilitation (n = 140)	26 (18.6)
Duration [days], median (range)	15.0 (1.0–120.0)
Hospitalization related to TGCT	151 (91.0)
≥ 5 hospitalizations	15 (9.9)
Duration [days], median (range)	3.0 (1.0–184.0)
Changed employment status from full-employment due to TGCT (n = 146)	17 (11.6)
Part-time employed	5 (3.4)
Unemployed	9 (6.2)
Retired	3 (2.1)
Work missed in 24 months prior to baseline (n = 116)	66 (56.9)
If work missed, number of [days], Median (range)	25.0 (1.0–75.0)
Domestic help required at baseline (n = 162)	26 (16.0)

*GP* general practitioner, *PT* physical therapy, *TGCT* tenosynovial giant cell tumor

While designed to report on epidemiologic data on dt-TGCT, the TOPP study is exposed to potential selection bias, (i.e., underreferral of less severe cases to tertiary sarcoma centers). In addition, patients referred to such sarcoma centers are generally more impaired by dt-TGCT, and the lack of patients treated in non-specialized centers could give an overestimation of the disease burden and healthcare utilization. To avoid selection of patients and thus violation of the “real-life” principle, no explicit non-eligibility criteria were defined. In addition, as data about medical history that were not considered essential or were difficult to remember were collected at baseline, an underreporting of data might have occurred.

The present findings from baseline and 2 years prior to study entry provide new insights into patient management before arriving in a tertiary sarcoma center. They strongly suggest that dt-TGCT has its onset in a relatively young and working population but whose dt-TGCT diagnosis is often delayed, most likely due to disease unfamiliarity or misdiagnosis. Evaluation of patient groups stratified by treatment received prior to study entry and at baseline in particular surgery and/or systemic therapy illustrate significant continued burden of disease. This is compounded by health economics and PRO data. Choice of treatment in the study population was mostly based on admission status, clinical experience, and PRO. Synovectomies were the mainstay of treatment, whereas TKIs were mostly restricted to severe and refractory cases, while a wait-and-see policy was applied for patients with less severe symptomatology. Within the context of these findings, developing multidisciplinary guidelines for the treatment of primary and refractory cases is of the utmost importance. Final results from the completed study will build upon these preliminary yet foundational understandings of the typical dt-TGCT patient journey profile in this rare disease.

## Supplementary Information

**Additional file 1**. TOPP registry description, breakdown by country, participating centers, and patient-reported outcome measurements.

## Data Availability

De-identified individual participant data (IPD) and applicable supporting clinical trial documents may be available upon request at https://vivli.org/ourmember/daiichi-sankyo/.
