# Supplementary material for: The diffuse-type tenosynovial giant cell tumor (dt-TGCT) patient journey: a prospective multicenter study
Source: Orphanet J Rare Dis. 2021 Apr 29;16:191. doi: 10.1186/s13023-021-01820-6 (PMC8086070; doi:10.1186/s13023-021-01820-6)
Supplement: Supplementary file 1 — Additional file 1. TOPP registry description, breakdown by country, participating centers, and patient-reported outcome measurements. [file 13023_2021_1820_MOESM1_ESM.docx]

## Tenosynovial Giant Cell Tumor Observational Platform Project (TOPP)

This is a multinational, multicenter, prospective, non-interventional observational disease registry. The sites will be specialized sites that treat TGCT regularly; no referral sites will be used, located in Austria, France, Germany, Italy, the Netherlands, Spain, the United Kingdom, and the United States of America.

| **Country** | **Patients (n = 166)** |
| --- | --- |
| Austria | 8 (4.8%) |
| France | 4 (2.4%) |
| Germany | 13 (7.8%) |
| Italy | 40 (24.1%) |
| The Netherlands | 60 (36.1%) |
| Spain | 8 (4.8%) |
| United Kingdom | 3 (1.8%) |
| United States | 30 (18.1%) |

## Participating centers

Department of Orthopaedics and Trauma, Medical University of Graz, Graz, Austria

Centre Hospitalier Universitaire de Nantes, Nantes, France

West deutschen Tumorzentrum, Essen, Germany

Fondazione IRCCS Istituto Nazionale dei Tumori, Milan, Italy

IRCCS Istituto Ortopedico Rizzoli, Bologna, Italy

Radboud University Medical Center, Nijmegen, The Netherlands

Leiden University Medical Center, Leiden, The Netherlands

University Castilla-La Mancha, Talavera de la Reina, Toledo, Spain

Hospital Universitario Virgen del Rocio, Sevilla, Spain

Oxford University Hospitals, Oxford, United Kingdom

UCLA Health, UCLA Medical Center, Santa Monica, US

Memorial Sloan Kettering Cancer Center, New York, US

## Patient-reported outcome measurements

TGCT-related patient-reported outcomes (PROs) collected at baseline were pain, stiffness, swelling, and limited range of motion. Relevant patient-reported outcome measurements (PROMs) administered at baseline consisted of the mean brief pain inventory (BPI), mean worst pain and stiffness numerical rating scale (NRS), Patient-Reported Outcome Measurement Information System Physical Functioning® (PROMIS-PF), and EuroQol 5D (EQ-5D). The BPI assesses the severity and interference of pain on a scale from 0 (not severe/no interference) to 10 (severe/complete interference) [1]. The worst pain and stiffness were scored on an NRS, requiring the patient to rate their worst pain and stiffness from 0 (nothing) to 10 (worst) in the last 24 hours. The PROMIS-PF evaluates physical, mental, and social health relevant to the affected limb region (upper/lower) with a score of 50 as mean in a US reference population [2]. The EQ-5D is a simple generic instrument and it is defined in terms of 5 dimensions: mobility, self-care, everyday activities, pain/discomfort, and anxiety/depression. The values or utilities are indicated on a scale where 0 corresponds to death and 1 corresponds to perfect health, with negative values also being possible. The second part of the EQ-5D consists of a vertical 20-cm, 0–100 visual analogue scale (VAS), where 0 represents the worst imaginable health state and 100 represents the best imaginable health state. The respondent marks a point on the scale to reflect their overall health on the day of the interview [3].

**References**

1. Cleeland CS, Gonin R, Hatfield AK, et al. Pain and its treatment in outpatients with metastatic cancer. N Engl J Med. 1994;330(9):592-6.

2. HealthMeasures. PROMIS 2019 [cited 2019 24 October 2019]. Available from: <http://www.healthmeasures.net/score-and-interpret/interpret-scores/promis>.

3. Dolan P. Modeling valuations for EuroQol health states. Med Care. 1997;35(11):1095-108.
